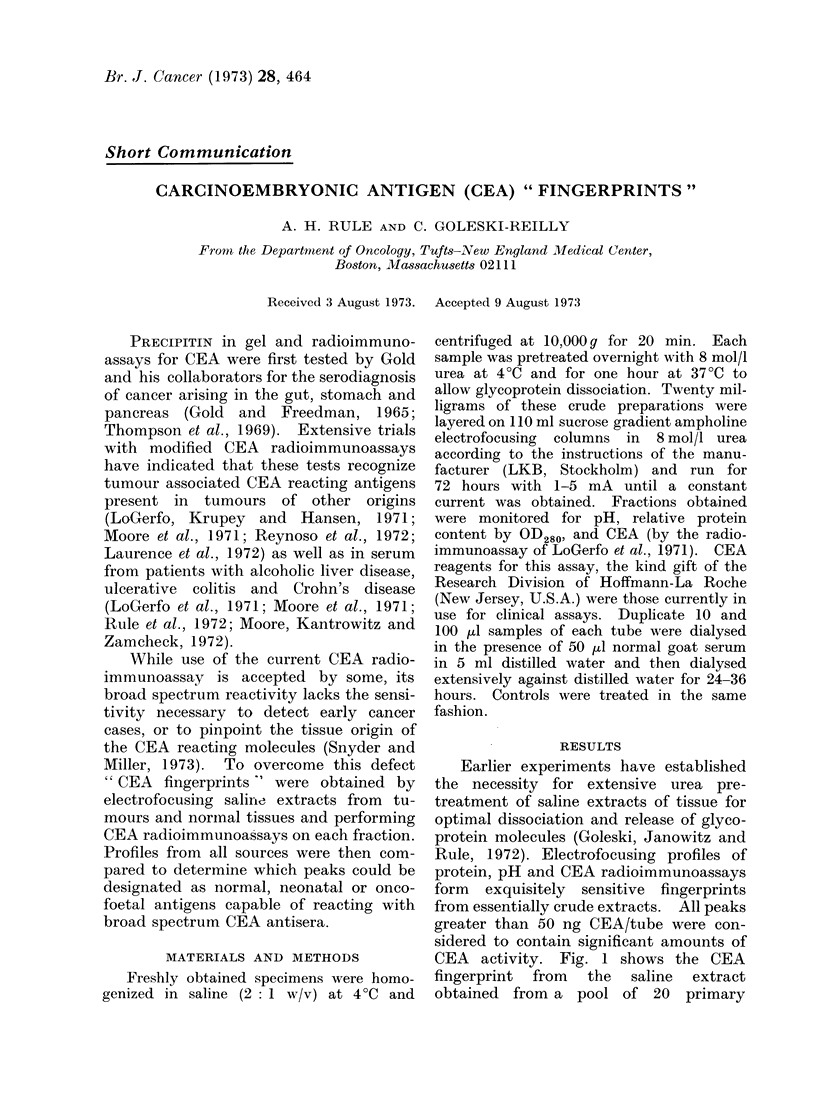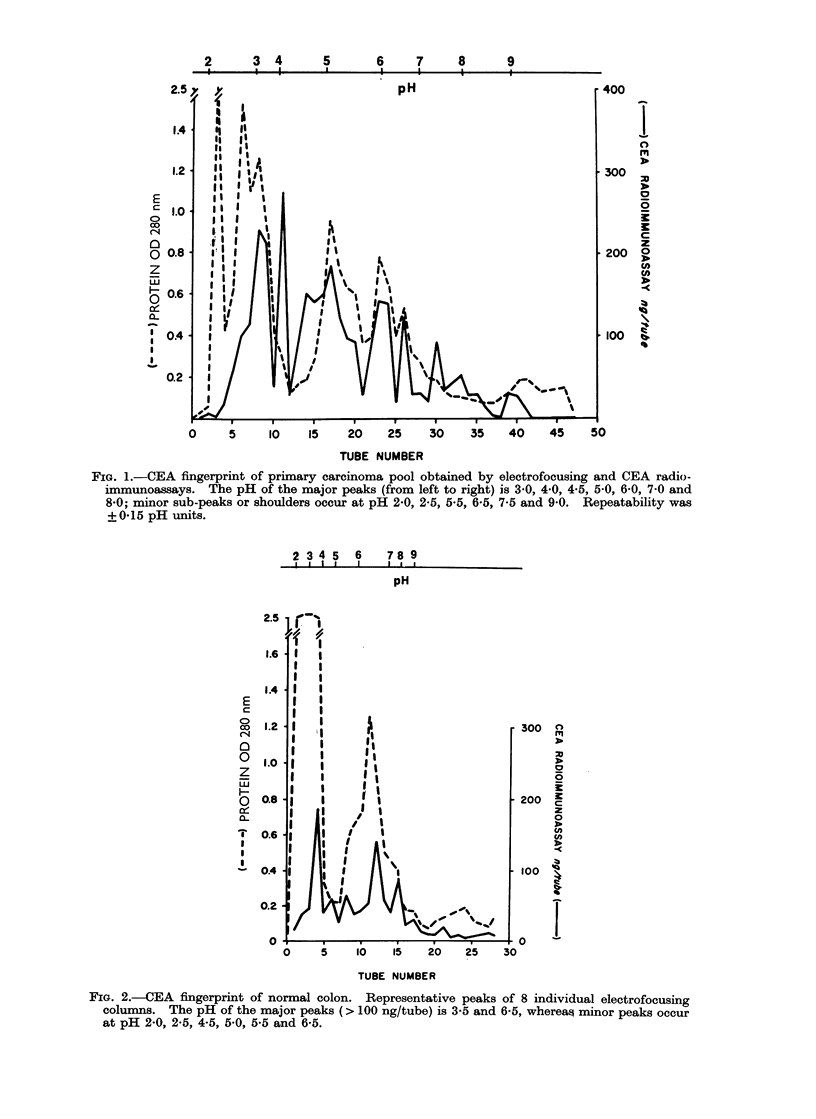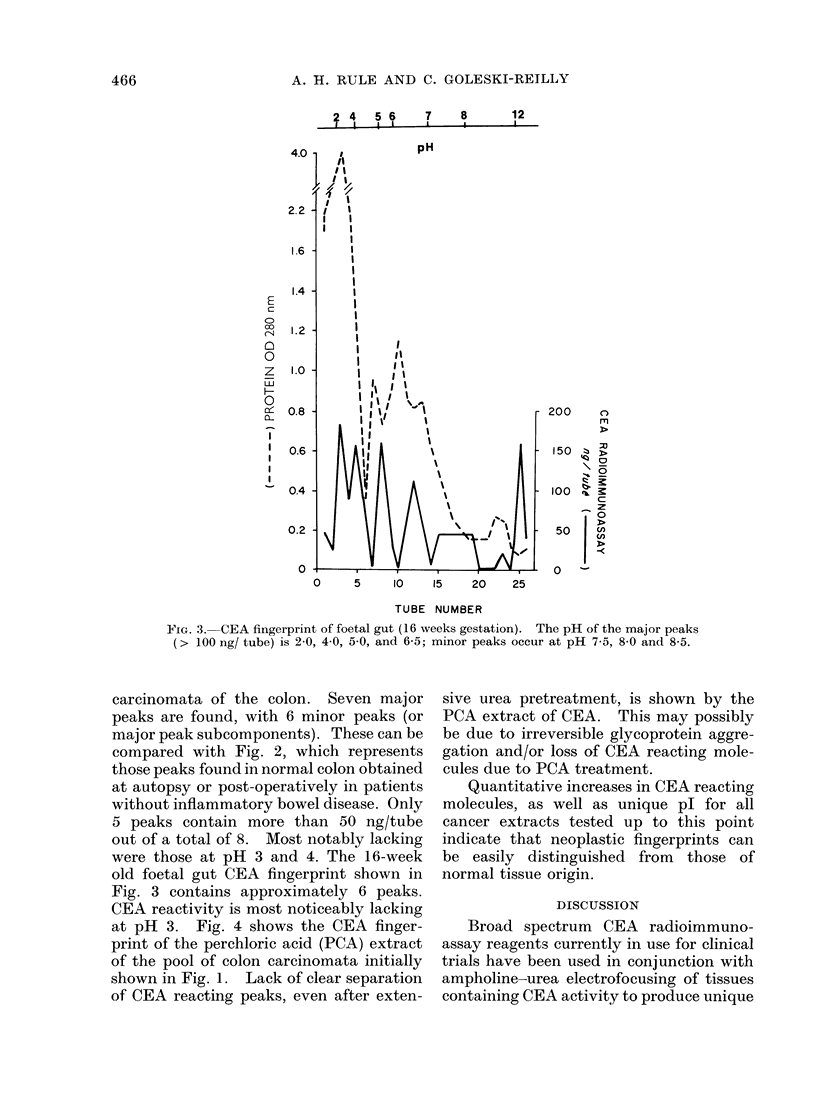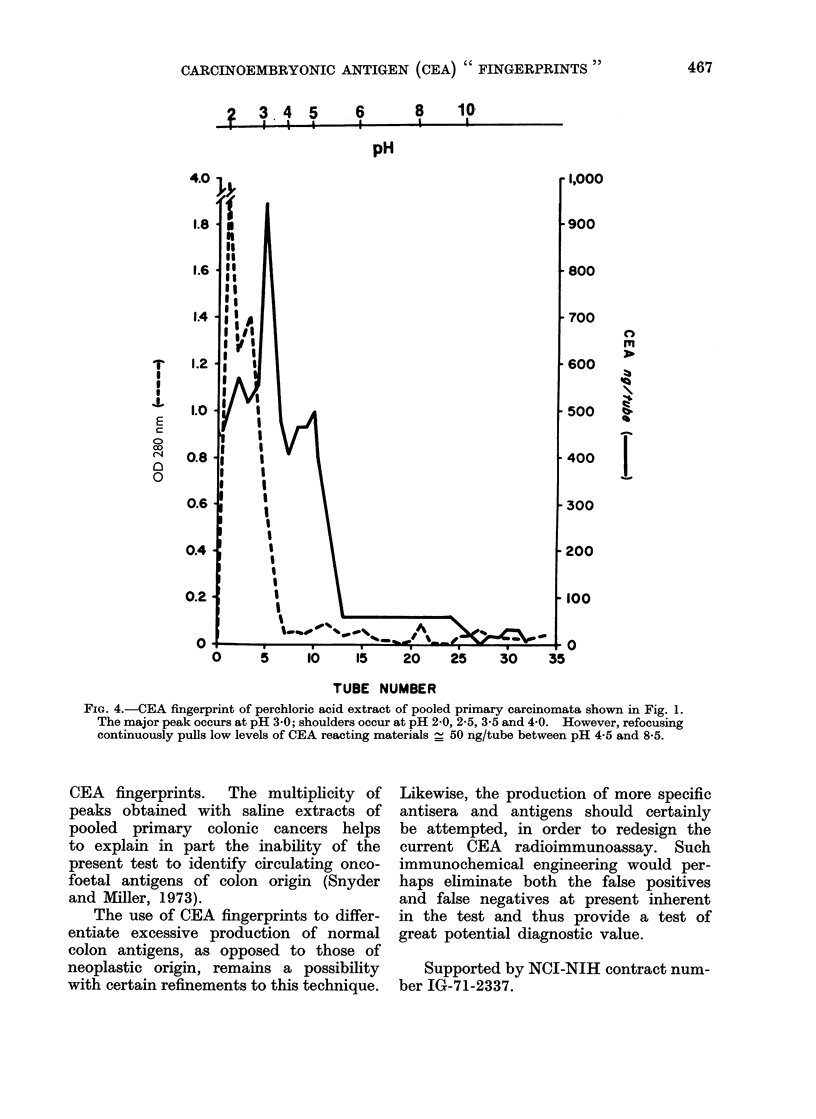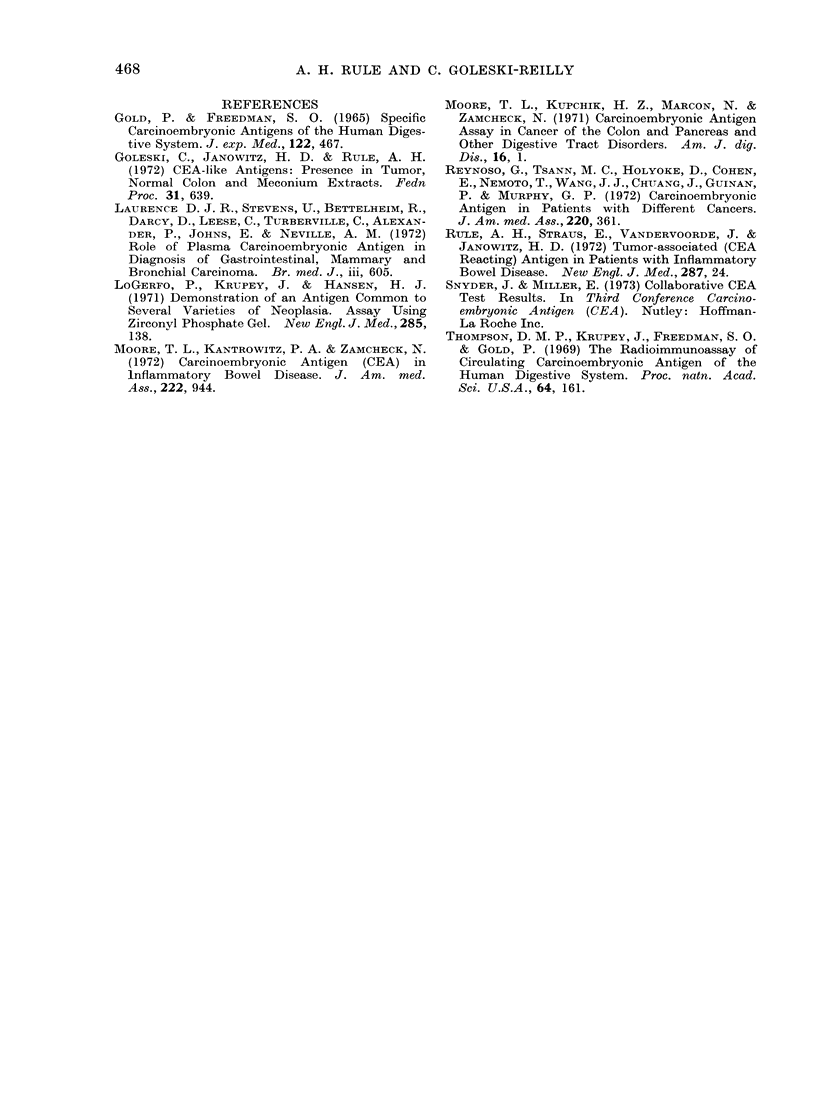# Carcinoembryonic antigen (CEA) "fingerprints".

**DOI:** 10.1038/bjc.1973.174

**Published:** 1973-11

**Authors:** A. H. Rule, C. Goleski-Reilly


*Br. J. Cancer* (1973) **28**, 464

***Short Communication***

### CARCINOEMBRYONIC ANTIGEN (CEA) " FINGERPRINTS "

A. H. RULE AND C. GOLESKI-REILLY

*From the Department of Oncology, Tufts–New England Medical Center, Boston, Massachusetts* 02111

Received 3 August 1973. Accepted 9 August 1973

PRECIPITIN in gel and radioimmunoassays for CEA were first tested by Gold and his collaborators for the serodiagnosis of cancer arising in the gut, stomach and pancreas (Gold and Freedman, 1965; Thompson *et al.*, 1969). Extensive trials with modified CEA radioimmunoassays have indicated that these tests recognize tumour associated CEA reacting antigens present in tumours of other origins (LoGerfo, Krupey and Hansen, 1971; Moore *et al.*, 1971; Reynoso *et al.*, 1972; Laurence *et al.*, 1972) as well as in serum from patients with alcoholic liver disease, ulcerative colitis and Crohn's disease (LoGerfo *et al.*, 1971; Moore *et al.*, 1971; Rule *et al.*, 1972; Moore, Kantrowitz and Zamcheck, 1972).

While use of the current CEA radioimmunoassay is accepted by some, its broad spectrum reactivity lacks the sensitivity necessary to detect early cancer cases, or to pinpoint the tissue origin of the CEA reacting molecules (Snyder and Miller, 1973). To overcome this defect " CEA fingerprints " were obtained by electrofocusing saline extracts from tumours and normal tissues and performing CEA radioimmunoassays on each fraction. Profiles from all sources were then compared to determine which peaks could be designated as normal, neonatal or oncofoetal antigens capable of reacting with broad spectrum CEA antisera.

### MATERIALS AND METHODS

Freshly obtained specimens were homogenized in saline (2 : 1 w/v) at 4°C and centrifuged at 10,000 $g$ for 20 min. Each sample was pretreated overnight with 8 mol/l urea at 4°C and for one hour at 37°C to allow glycoprotein dissociation. Twenty milligrams of these crude preparations were layered on 110 ml sucrose gradient ampholine electrofocusing columns in 8 mol/l urea according to the instructions of the manufacturer (LKB, Stockholm) and run for 72 hours with 1–5 mA until a constant current was obtained. Fractions obtained were monitored for pH, relative protein content by $OD_{280}$, and CEA (by the radioimmunoassay of LoGerfo *et al.*, 1971). CEA reagents for this assay, the kind gift of the Research Division of Hoffmann-La Roche (New Jersey, U.S.A.) were those currently in use for clinical assays. Duplicate 10 and 100 $\mu$l samples of each tube were dialysed in the presence of 50 $\mu$l normal goat serum in 5 ml distilled water and then dialysed extensively against distilled water for 24–36 hours. Controls were treated in the same fashion.

### RESULTS

Earlier experiments have established the necessity for extensive urea pretreatment of saline extracts of tissue for optimal dissociation and release of glycoprotein molecules (Goleski, Janowitz and Rule, 1972). Electrofocusing profiles of protein, pH and CEA radioimmunoassays form exquisitely sensitive fingerprints from essentially crude extracts. All peaks greater than 50 ng CEA/tube were considered to contain significant amounts of CEA activity. Fig. 1 shows the CEA fingerprint from the saline extract obtained from a pool of 20 primary

FIG. 1.—CEA fingerprint of primary carcinoma pool obtained by electrofocusing and CEA radioimmunoassays. The pH of the major peaks (from left to right) is 3·0, 4·0, 4·5, 5·0, 6·0, 7·0 and 8·0; minor sub-peaks or shoulders occur at pH 2·0, 2·5, 5·5, 6·5, 7·5 and 9·0. Repeatability was ±0·15 pH units.

FIG. 2.—CEA fingerprint of normal colon. Representative peaks of 8 individual electrofocusing columns. The pH of the major peaks (> 100 ng/tube) is 3·5 and 6·5, whereas minor peaks occur at pH 2·0, 2·5, 4·5, 5·0, 5·5 and 6·5.

FIG. 3.—CEA fingerprint of foetal gut (16 weeks gestation). The pH of the major peaks (> 100 ng/ tube) is 2·0, 4·0, 5·0, and 6·5; minor peaks occur at pH 7·5, 8·0 and 8·5.

carcinomata of the colon. Seven major peaks are found, with 6 minor peaks (or major peak subcomponents). These can be compared with Fig. 2, which represents those peaks found in normal colon obtained at autopsy or post-operatively in patients without inflammatory bowel disease. Only 5 peaks contain more than 50 ng/tube out of a total of 8. Most notably lacking were those at pH 3 and 4. The 16-week old foetal gut CEA fingerprint shown in Fig. 3 contains approximately 6 peaks. CEA reactivity is most noticeably lacking at pH 3. Fig. 4 shows the CEA fingerprint of the perchloric acid (PCA) extract of the pool of colon carcinomata initially shown in Fig. 1. Lack of clear separation of CEA reacting peaks, even after extensive urea pretreatment, is shown by the PCA extract of CEA. This may possibly be due to irreversible glycoprotein aggregation and/or loss of CEA reacting molecules due to PCA treatment.

Quantitative increases in CEA reacting molecules, as well as unique pI for all cancer extracts tested up to this point indicate that neoplastic fingerprints can be easily distinguished from those of normal tissue origin.

### DISCUSSION

Broad spectrum CEA radioimmunoassay reagents currently in use for clinical trials have been used in conjunction with ampholine–urea electrofocusing of tissues containing CEA activity to produce unique

FIG. 4.—CEA fingerprint of perchloric acid extract of pooled primary carcinomata shown in Fig. 1. The major peak occurs at pH 3·0; shoulders occur at pH 2·0, 2·5, 3·5 and 4·0. However, refocusing continuously pulls low levels of CEA reacting materials ≅ 50 ng/tube between pH 4·5 and 8·5.

CEA fingerprints. The multiplicity of peaks obtained with saline extracts of pooled primary colonic cancers helps to explain in part the inability of the present test to identify circulating oncofoetal antigens of colon origin (Snyder and Miller, 1973).

The use of CEA fingerprints to differentiate excessive production of normal colon antigens, as opposed to those of neoplastic origin, remains a possibility with certain refinements to this technique. Likewise, the production of more specific antisera and antigens should certainly be attempted, in order to redesign the current CEA radioimmunoassay. Such immunochemical engineering would perhaps eliminate both the false positives and false negatives at present inherent in the test and thus provide a test of great potential diagnostic value.

Supported by NCI-NIH contract number IG-71-2337.

REFERENCES

GOLD, P. & FREEDMAN, S. O. (1965) Specific Carcinoembryonic Antigens of the Human Digestive System. *J. exp. Med.*, **122**, 467.

GOLESKI, C., JANOWITZ, H. D. & RULE, A. H. (1972) CEA-like Antigens: Presence in Tumor, Normal Colon and Meconium Extracts. *Fedn Proc.* **31**, 639.

LAURENCE D. J. R., STEVENS, U., BETTELHEIM, R., DARCY, D., LEESE, C., TURBERVILLE, C., ALEXANDER, P., JOHNS, E. & NEVILLE, A. M. (1972) Role of Plasma Carcinoembryonic Antigen in Diagnosis of Gastrointestinal, Mammary and Bronchial Carcinoma. *Br. med. J.*, iii, 605.

LOGERFO, P., KRUPEY, J. & HANSEN, H. J. (1971) Demonstration of an Antigen Common to Several Varieties of Neoplasia. Assay Using Zirconyl Phosphate Gel. *New Engl. J. Med.*, **285**, 138.

MOORE, T. L., KANTROWITZ, P. A. & ZAMCHECK, N. (1972) Carcinoembryonic Antigen (CEA) in Inflammatory Bowel Disease. *J. Am. med. Ass.*, **222**, 944.

MOORE, T. L., KUPCHIK, H. Z., MARCON, N. & ZAMCHECK, N. (1971) Carcinoembryonic Antigen Assay in Cancer of the Colon and Pancreas and Other Digestive Tract Disorders. *Am. J. dig. Dis.*, **16**, 1.

REYNOSO, G., TSANN, M. C., HOLYOKE, D., COHEN, E., NEMOTO, T., WANG, J. J., CHUANG, J., GUINAN, P. & MURPHY, G. P. (1972) Carcinoembryonic Antigen in Patients with Different Cancers. *J. Am. med. Ass.*, **220**, 361.

RULE, A. H., STRAUS, E., VANDERVOORDE, J. & JANOWITZ, H. D. (1972) Tumor-associated (CEA Reacting) Antigen in Patients with Inflammatory Bowel Disease. *New Engl. J. Med.*, **287**, 24.

SNYDER, J. & MILLER, E. (1973) Collaborative CEA Test Results. In *Third Conference Carcinoembryonic Antigen* (*CEA*). Nutley: Hoffman-La Roche Inc.

THOMPSON, D. M. P., KRUPEY, J., FREEDMAN, S. O. & GOLD, P. (1969) The Radioimmunoassay of Circulating Carcinoembryonic Antigen of the Human Digestive System. *Proc. natn. Acad. Sci. U.S.A.*, **64**, 161.